# Safety and feasibility of directional coronary atherectomy with transradial approach using an 8Fr sheathless guiding catheter

**DOI:** 10.1038/s41598-025-11349-4

**Published:** 2025-07-14

**Authors:** Tsuyoshi Ota, Takahiro Sawada, Masahiro Koide, Masamichi Iwasaki, Koichi Nakamura, Yoichiro Matsuoka, Yuya Terao, Tatsuro Ito, Takeaki Shirai, Katsunori Okajima, Makoto Kadotani, Yoshio Onishi, Ken-ichi Hirata

**Affiliations:** 1 Department of Cardiology, Kakogawa Central City Hospital, 439 Honmachi, Kakogawacho-Honmachi, Kakogawa, 675-8611 Hyogo Japan; 2 Department of Cardiology, Kyoto Okamoto Memorial Hospital, Kyoto, Japan; 3 Department of Cardiology, Hyogo Prefectural Awaji Medical Center, Sumoto, Japan

**Keywords:** Directional coronary atherectomy, Transradial, Vascular access site complication, Cardiology, Interventional cardiology

## Abstract

This study aimed to assess the safety and feasibility of the transradial approach (TRA) using the 8Fr sheathless guiding catheter (GC) in directional coronary atherectomy (DCA). This retrospective analysis included all consecutive patients who underwent percutaneous coronary intervention with DCA from April 2021 to March 2024 in three cardiovascular centers. During the study period, 194 DCA procedures were performed. Of these, 51 included the TRA using an 8Fr sheathless guiding system, whereas the remaining 143 included the conventional transfemoral approach (TFA) system. Primary outcomes were the vascular access site complication (VASC), defined as a bleeding complication in the perioperative period, retroperitoneal hematoma, pseudoaneurysm, large hematoma around the puncture site, or access vessel occlusion rate, and technical success rates, defined as delivery of the DCA catheter to the target lesion and plaque debulking to < 60% of the %plaque area. Although there were some bias such as the lower lesion complexity in the TRA group, the VASC rate was significantly lower in the TRA group than in the TFA group (*p* = 0.02) and both approaches had > 80% technical success rates. In conclusion, DCA with TRA using an 8Fr sheathless GC may be a safe and feasible method.

## Introduction

A directional coronary atherectomy (DCA) catheter (Atherocut™, NIPRO, Japan) is used to remove atherosclerotic plaques in the coronary artery during percutaneous coronary intervention (PCI). Although the sale of the DCA catheter was initially discontinued following the development of the drug-eluting stent (DES), it was re-introduced in Japan in 2014. Since then, many studies have reported the effectiveness of the DCA catheter, particularly in Japan^1–3^. DCA could avoid complex stenting and improve the treatment outcomes with first-generation DES for bifurcated lesions owing to the prevention of plaque and carina shift by debulking the plaque volume^4^. Furthermore, successful plaque removal by DCA provides sufficient vessel lumen. Accordingly, stent implantation was no longer necessary, and the procedure might be terminated with a drug-coated balloon (DCB) alone^5^. Therefore, high bleeding risk, younger patients who did not want stent implantation, and patients with bifurcated lesions, such as ostial lesions of the left anterior descending branch are considered to be good candidates for DCA.

Although the re-introduced DCA catheter can be inserted into the 7Fr guiding catheter (GC), it is quite tight for the 7Fr GC, and the direction of the long axis is difficult to obtain angiographically. Therefore, the 8Fr GC is recommended for accurately confirming the debulking position for DCA, and this is generally performed using the transfemoral approach (TFA)^2^. In the 2000 s, the TFA was the preferred access site approach for PCI because the larger-sized GCs could be used. When larger-sized GCs are used, they provide a stronger back-up force, making PCI easier and increasing the options available for device choice. However, since the ACCESS study, which was the first randomized control study to compare the transradial approach (TRA) with other access site approaches, many studies have demonstrated that TRA reduced not only complications at the puncture site but also ischemic events compared with the TFA, and a paradigm shift has occurred regarding access site for PCI^6–8^. Recently, Niizeki et al. reported on the usefulness of the TRA using the 8Fr sheathless GC in DCA^9^. However, research on the incidence of procedure failure and vascular complications of these methods is limited. This study aimed to retrospectively assess the complication rates and the procedural success in the patients who underwent DCA with the TRA using the 8Fr sheathless GC.

## Materials and methods

### Study design and participants

This was a non-randomized, retrospective observational study that included all consecutive patients who underwent DCA from April 2021 to March 2024 at three cardiovascular centers: Kakogawa Central City Hospital, Kyoto Okamoto Memorial Hospital, and Hyogo Prefectural Awaji Medical Center. Because this study was conducted retrospectively from data obtained for clinical purpose, all procedures being performed were part of routine care.

*The Ethics Committee of Kakogawa Central City Hospital approved this study (Approval No. 2024-50). In other participating institutions where formal ethical review was not required due to the limited number of cases and the use of anonymized*,* retrospective data*,* the study was conducted in accordance with institutional regulations and the principles of the Declaration of Helsinki.* Informed consent was obtained from individual participants included in the study after explaining the study objectives and methodology. Patients signed informed consent regarding publishing their data. All methods were performed in accordance with the relevant guidelines and regulations.

We assessed the patient characteristics, including age, sex, height, body weight, body mass index, presence of comorbidities (dialysis, hypertension, dyslipidemia, diabetes mellitus, and smoking history), coronary artery disease presentation (stable angina or acute coronary syndrome [ACS]) and use of antithrombotic medications (antiplatelet agents and anticoagulant drugs, dual or triple antiplatelet therapy).

Angiographically, we assessed treated vessel and traditional lesion types using the American Heart Association/American College of Cardiology’s classification, the location of the lesion (proximal, mid, or distal vessel) and whether chronic total occlusion occurred. A coronary cine angiogram was performed after intracoronary injection of nitroglycerin (250 µg), and the severity of stenosis was measured using a quantitative coronary arteriography cardiovascular measurement system (CMS-Medis Medical Imaging Systems, Leiden, the Netherlands).

### PCI and DCA procedures

The access strategy (TRA or TFA) was chosen based on feasibility, including radial artery size, lesion characteristics, and operator discretion.

All patients were pretreated with aspirin 100 mg and prasugrel 3.75 mg at least 14 days before the procedure, or a loading dose of 20 mg was administered immediately before PCI. Precisely, 100 U/kg of unfractionated heparin was injected intravenously to maintain an activated clotting time > 250 s during the procedure. PCI was performed using the routinely used 8Fr GC, such as JCL4.0 (Launcher™, Medtronic, Minneapolis, MN, USA) or CL4.0 (RoadMaster™, GOODMAN, Aichi, Japan) for the left coronary artery and JR4.0 (Launcher, Medtronic) for the right coronary artery. All procedures were performed under the supervision of a surgeon with over 15 years of PCI experience and at least 10 cases of prior DCA experience, but no prior experience with TRA using an 8Fr sheathless GC (T.S., M.K., M.K., or M.I.).

The TRA procedure was performed as follows: the right or left radial artery was punctured after local anesthesia with 1% lidocaine, using the blind puncture technique. A 6Fr Glidesheath Slender™ (Terumo Medical Corporation, Tokyo, Japan) was introduced. As previously reported by Niizeki, the 8Fr sheathless GC was made by inserting the 125-cm 6.5Fr tapered tip inner catheter (STA, MEDKIT Co. Ltd., Tokyo, Japan) into a 100-cm 8Fr GC. After inserting the 6Fr sheath, we exchanged using a 260-cm 0.035-inch stiff 1.5 J guidewire (Terumo Medical Corporation) from the 6Fr sheath to the 8Fr sheathless GC^9^. The entire system was advanced to the ascending aorta, and the 8Fr GC was engaged into the coronary ostium after removing the inner catheter. Although this sheathless method was not specifically tailored for radial artery intervention, this was very similar to the previously reported Railtracking technique, which was explicitly designed to optimize compatibility, precision, and ease of use for TRA procedures^10^. After the procedure, the radial puncture site was compressed with an Air-Band Radial Compression Device (TR band™, Terumo Medical Corporation).

The TFA procedure was performed as follows: the common femoral artery was punctured under ultrasound guidance and a 40-cm 8Fr long sheath (Terumo Medical Corporation) was inserted. The 8Fr GC was inserted from the 8Fr sheath in a similar fashion to TRA. The femoral puncture site was closed with either manual compression or a closure device (Perclose ProGlide™; Abbott, Chicago, USA or Angioseal™; Terumo Medical Corporation) depending on the discretion of the main surgeon.

The indications for DCA were determined after intravascular ultrasound (IVUS) or optical frequency domain imaging (OFDI) evaluation. Briefly, they were vessel diameter > 3.5 mm and the absence of maximum superficial calcification angle > 270° that might interfere with DCA delivery. DCA was performed routinely, typically started by cutting with low balloon pressures. Subsequently, multiple cuts were performed using gradually increasing balloon pressures under IVUS or OFDI guidance. IVUS and OFDI images were examined at baseline and post-DCA. We measured the lumen cross sectional area (CSA), vessel CSA, and plaque area. Percent plaque area (%PA) was defined as the plaque area divided by vessel CSA at minimum lumen site. In line with the previous studies, the goal of the optimal DCA procedure was less than 60% of %PA on IVUS and OFDI imaging^3,11^. If plaque resection was considered challenging due to superficial calcification, which met the indication for DCA, then rotablator of orbital atherectomy was performed before DCA. Following DCA, we finalized PCI through DES implantation, DCB dilation alone, or both DES and DCB, based on the surgeon’s discretion. In all cases, the procedure and fluoroscopy time, contrast volume, and radiation dosage were recorded. The procedure time was defined as the time from the start of puncture to the time of GC removal.

After the procedure was completed, patients were transferred to a clinical ward or intensive care unit for observation in case of ACS, and antiplatelet therapy was continued according to Japanese clinical guidelines.

### Primary outcomes

The primary outcomes were the vascular access site complications (VASC) rate and the technical success rate. We defined VASC as a bleeding complication in the perioperative period, retroperitoneal hematoma, pseudoaneurysm, large hematoma around the puncture site, or access vessel occlusion. We defined bleeding complications as follows: clinically overt signs of hemorrhage associated with a > 3 g/dL drop in hemoglobin or > 10% absolute decrease in hematocrit reduction during the perioperative period^12,13^. Large hematoma around the puncture site was defined as that documented in the nursing record, which was > 5 cm subcutaneous swelling around the puncture site^13^. Radial artery occlusion was assessed by doppler ultrasound or radial pulse palpation at 24 h post-procedure or at discharge, according to institutional practice. Technical success was defined as delivery of the DCA catheter to the target lesion and plaque debulking to less than 60% of the %PA.

### Statistical methods

All the statistical analyses were performed using Medcalc (version 20.023; Medcalc Software, Mariakerke, Belgium).

Baseline procedure characteristics are expressed as number and percentage for dichotomous variables or as mean and standard deviation for continuous variables with normal distribution, else as median and interquartile ranges. Differences in VASC and the technical success rates between the different approaches were determined using the chi-square test. Statistical significance was defined as *P* < 0.05. Comparison of continuous variables between the two groups was performed using the unpaired t-test. If the data were not normally distributed, as determined using the Kolmogorov–Smirnov analysis, then the data were analyzed using the Mann–Whitney test.

## Results

During the study period, 200 DCA procedures were performed in the three institutions. Eight patients underwent the procedure twice for other vessel intervention (2 patients; TRA + TRA, 5 patients; TFA + TFA, 1 patient, TRA + TFA). We excluded six procedures who underwent DCA via the distal radial artery. Among the 194 DCA procedures, 51 were allocated to the TRA group and the remaining 143 were in the TFA group. The patient characteristics are shown in Table 1. Considering the patient characteristics, younger and heavier patients were included in the TRA group compared to those in the TFA group. Eleven patients in the TFA group and none in the TRA group underwent dialysis. No significant differences were observed in other comorbidities and antithrombotic drugs between the two groups. For coronary artery disease presentation, the TRA group had more cases of ACS than the TFA group.

**Table 1:** Patient characteristics.

	TRA (*n* = 51)	TFA (*n* = 143)	*P* value
Sex (male, %)	47 (92.3%)	126 (88.1%)	0.43
Age (years)	64.1 ± 11.8	68.4 ± 10.8	0.02
Height (cm)	168.2 ± 8.7	165.9 ± 7.8	0.08
Body weight (kg)	71.1 ± 16.7	66.1 ± 11.5	0.02
Body mass index(kg/m2)	24.8 (21.8, 27.9)	23.9 (21.8, 26.1)	0.20
*Comorbidities*			
Dialysis (n, %)	0 (0%)	11 (7.7%)	0.04
Hypertension (n, %)	42 (82.4%)	114 (79.7%)	0.69
Dyslipidemia (n, %)	40 (78.4%)	121 (84.6%)	0.31
Current smoker (n, %)	38 (74.5%)	79 (55.2%)	0.02
Diabetes mellitus (n, %)	25 (49.0%)	63 (44.1%)	0.54
*CAD presentation*			
Stable angina (n, %)	33 (64.7%)	121 (84.6%)	
Acute coronary syndrome (n, %)	18 (35.3%)	22 (15.4%)	0.0026
*Antithrombotic drug*			
Aspirin (n, %)	50 (98.0%)	137 (93.7%)	
Thienopyridine (n, %)	51 (100%)	141 (98.6%)	
Warfarin (n, %)	2 (3.9%)	1 (0.7%)	
Direct oral anticoagulant (n, %)	2 (3.9%)	9 (6.3%)	0.61
Dual antiplatelet therapy (n, %)	47 (92.1%)	131 (91.6%)	
Triple antiplatelet therapy (n, %)	3 (5.9%)	4 (2.8%)	0.61

TRA: trans radial approach, TFA: trans femoral approach, CAD: coronary artery disease.

As shown in Table 2, there were no significant differences in the quantitative coronary angiography data between the two groups. In the TRA group, the right and left side approaches had 31 (60.8%) and 20 (39.2%), respectively. Although the frequency of M size DCA catheter use was significantly higher in the TRA group than that in the TFA group, there were no significant differences in other DCA procedures between the two groups. The lower lesion complexity in the TRA group may results in less radiation, less fluoroscopy and less procedure time. In both groups, lumen CSA was significantly increased and plaque area and %PA were significantly decreased. Median %PA after DCA was similar between the two groups (53.9% in the TRA group vs. 54.2% in the TFA group).

**Table 2:** Lesion characteristics and percutaneous coronary intervention procedure.

	TRA (*n* = 51)	TFA (*n* = 143)	*P* value
*Disease vessel*			
Left main trunk/Left anterior descending artery (n, %)	38 (74.5%)	122 (85.3%)	
Left circumflex artery (n, %)	4 (7.8%)	5 (3.5%)	
Right coronary artery (n,%)	9 (17.6%)	16 (11.2%)	0.12
*Type of lesion*			
B2/C (n, %)	37 (72.5%)	124 (86.7%)	0.02
*Location of lesion*			
Proximal/Mid (n, %)	38 (74.5%)/13 (25.5%)	119 (83.2%)/24 (16.8%)	0.18
Chronic total occlusion (n, %)	3 (5.9%)	16 (11.2%)	0.27
*QCA data*			
**Pre**			
Lesion length (mm)	18.9 (10.6, 24.9)	20.3 (16.9, 29.4)	0.12
Reference vessel diameter (mm)	3.17 (3.03, 3.89)	3.02 (2.46, 3.63)	0.18
Minimum lumen diameter (mm)	1.10 (0.47, 1.39)	0.77 (0.35, 1.11)	0.22
%diameter stenosis (%)	65.3 (60.7, 87.1)	73.3 (60.5, 85.1)	0.62
**Post**			
Minimum lumen diameter (mm)	2.83 (2.59, 3.07)	2.83 (2.56, 3.15)	0.93
%diameter stenosis (%)	14.0 (10.1, 20.0)	11.4 (8.9, 18.6)	0.47
Acute gain (mm)	1.89 (1.45, 2.60)	2.06 (1.65, 2.58)	0.39
Use of IVUS (n, %)	38 (74.5%)	128 (89.5%)	0.01
*IVUS and OFDI measurements*			
**Pre**			
Lumen cross sectional area (mm^2^)	2.6 (2.0, 3.7)	2.9 (2.2, 3.8)	0.47
Vessel cross sectional area (mm^2^)	16.5 (13.1, 20.3)	15.3 (12.3, 17.7)	0.11
Plaque area (mm^2^)	13.7 (11.0, 16.5)	12.0 (9.2, 14.7)	0.07
%Plaque area (%)	84.1 (77.4, 88.2)	80.1 (73.6, 85.9)	0.06
**Post**			
Lumen cross sectional area (mm^2^)	8.6 (7.0, 9.9)	7.8 (6.4, 9.2)	0.13
Vessel cross sectional area (mm^2^)	19.0 (15.0, 21.0)	16.7 (14.4, 20.4)	0.26
Plaque area (mm^2^)	9.1 (7.2, 12.1)	9.3 (7.4, 11.5)	0.57
%plaque area (%)	53.9 (45.6, 58.2)	54.2 (48.4, 59.4)	0.55
Use of rotablator/orbital atherectomy (n, %)	6 (11.8%)	37 (25.9%)	0.04
Approach side (right/left, n, %)	31 (60.8%)/20 (39.2%)		
*DCA catheter*			
L size (n, %)	30 (58.9%)	121 (84.6%)	0.001
Number of DCA sessions (n, %)	3 (2, 4)	3 (2, 4)	0.28
Number of DCA cutting (n, %)	11 (8, 16)	14 (10, 20)	0.06
DCA balloon pressure (atm)	3 (2, 4)	3 (2, 4)	0.06
*Finalize device*			
Drug-eluting stent (n, %)	5 (9.8%)	19 (13.3%)	
Drug-coated balloon (n, %)	43 (84.3%)	109 (76.2%)	
DES + DCB (n, %)	3 (5.9%)	15 (10.5%)	0.88
Procedure time (min)	101.0 (67.0, 120.0)	114.0 (88.5, 145.0)	0.002
Fluoroscopy time (min)	36.0 (28.2, 43.8)	46.0 (34.7, 58.7)	0.001
Contrast volume (ml)	148 (123, 168)	163 (120, 202)	0.07
Radiation dose (Gy)	1.9 (1.3, 3.5)	2.8 (1.9, 3.9)	0.009
Pre Hemoglobin level (mg/dL)	14.1 ± 1.6	13.6 ± 2.0	0.16
Post Hemoglobin Level (mg/dL)	13.3 ± 1.5	12.5 ± 1.9	0.007
Changes in Hemoglobin level (mg/dL)	0.7 (0.3, 1.0)	1.0 (0.4, 1.6)	0.003
Pre Hematocrit (%)	41.1 (38.5, 44.9)	41.7 (37.4, 44.9)	0.71
Post Hematocrit (%)	39.3 (36.4, 42.0)	37.5 (33.1, 41.0)	0.02
Changes in Hematocrit (%)	2.2 (0.8, 3.6)	3.5 (1.9, 5.9)	0.0006
*Method of hemostasis*			
Air band (n, %)	51 (100%)	0 (0%)	
Manual compression (n, %)	0 (0%)	50 (35.0%)	
Perclose (n, %)	0 (0%)	84 (58.7%)	
Angioseal (n, %)	0 (0%)	9 (6.3%)	

QCA: quantitative coronary angiography, DCA: directional coronary atherectomy, DES: drug-eluting stent, DCB: drug-coated balloon, TRA: trans radial approach, TFA: trans femoral approach.

All hemostasis methods used in the TRA group were performed using the Air-Band Radial Compression Device, whereas in the TFA group, manual compression was used for 35.0%, Perclose ProGlide for 58.7% and Angioseal for 6.3% of the patients. Although the hemoglobin level and hematocrit before PCI were similar between the two groups, reduction in the hemoglobin level and hematocrit after PCI in the TRA group was significantly lower than that in the TFA groups.

Table 3 shows primary outcomes, major adverse cardiac events of 24 h and 1 months and non-access site complication. There were eight cases of perioperative bleeding and two cases of pseudoaneurysm in the TFA group, but none of these were observed in the TRA group. No access vessel occlusion was observed in the TFA group; two patients showed radial artery occlusion in the TRA group. The VASC rate of the TRA group was significantly lower than that of the TFA group (3.9% vs. 16.8%, *P* = 0.02). The technical success rate of the TRA group was 80.4% and there was no difference in the approach side, whereas that of the TFA group was 81.1%, which was almost equal between the two groups. No crossover was required in any of the TRA and TFA cases.

**Table 3:** Primary outcomes, major adverse cardiac event and non-access cite complications.

	TRA (*n* = 51)	TFA (*n* = 143)	*P* value
*Primary outcomes*			
Vascular access site complication (n, %)	2 (3.9%)	24 (16.8%)	0.02
Perioperative bleeding (n, %)	0 (0%)	8 (5.6%)	0.08
Retroperitoneal hematoma (n, %)	0 (0%)	0 (0%)	0.99
Pseudo aneurysm (n, %)	0 (0%)	2 (1.4%)	0.40
Local large hematoma (n, %)	2 (3.9%)	16 (11.2%)	0.13
Access vessel occlusion (n, %)	2 (3.9%)	0 (0%)	0.09
Technical success (n, %)	41 (80.4%)	116 (81.1%)	0.85
Access site conversion (n, %)	0 (0%)	0 (0%)	0.99
Major adverse cardiac event (n, %)	2 (5.7%)	9 (7.8%)	0.68
After 24 h (n, %)	1 (2.9%)	0 (0%)	0.07
After 1 month (n, %)	2 (5.7%)	9 (7.8%)	0.68
Cerebral infarction (n, %)	1 (2.9%)	0 (0%)	0.07
Bacteremia (n, %)	0 (0%)	3 (2.6%)	0.34

TRA: trans radial approach, TFA: trans femoral approach.

Few non-access site complications occurred in both groups; one case of cerebral infarction in the TRA group and three cases of bacteremia in the TFA group.

During follow-up (all cases had a follow-up period of at least 1 month), one case of target vessel revascularization in the TRA group occurred within 24 h of the DCA procedure because the distal part of a DCA lesion occluded due to dissection by balloon angioplasty. Of 151 patients, four (3.4%) died. All of them had undergone DCA with the TFA. The causes of death included heart failure (*n* = 1), interstitial pneumonia (*n* = 1), and unknown (*n* = 2). None of these deaths were directly related to the procedure; e.g., one patient who underwent emergent PCI and DCA with the TFA under venoarterial extracorporeal membrane oxygenation (V-A ECMO) and Impella implantation for ST-elevated myocardial infarction with left main trunk obstruction died during hospitalization. The patient did not demonstrate hemodynamic improvement, was unable to wean from V-A ECMO and Impella, and died on day 23 of hospitalization.

## Discussion

To our knowledge, this is the first study to demonstrate that the TRA using the 8Fr sheathless GC is a safer and equally feasible method for DCA compared with the conventional TFA. In our cohort, the TRA group achieved a high technical success rate (80.4%) without any crossover, supporting the practical applicability of this technique in appropriately selected patients.

In the past 20 years, the TRA has been a routinely used approach instead of the TFA for PCI, and many studies have reported significantly lower complication rates of the TRA than those of the TFA, thus reducing the patient burden^6–8^. In rotational atherectomy, which is a method of coronary atherectomy for a longer period, compared with DCA, the TRA was associated with equivalent 30-day mortality and procedural success but reduced significant bleeding and access site complications when compared with the TFA^14^. However, regarding DCA, no data are available on whether the TRA is favorable or not due to limited GC sizes. Recently, two novel methods have been reported for performing DCA using the TRA. Numasawa et al.^15^ reported the dual catheter system via the bilateral radial arteries and Niizeki et al.^7^ reported an 8Fr TRA sheathless GC system. Although both methods were considered excellent ways to comfortably perform DCA, the dual catheter system needs another puncture site for secondary access and could lead to other VASC. Therefore, we employed the 8Fr TRA sheathless GC system to conduct this study. The external diameter of the 8Fr GC is equivalent to the external diameter of the 6Fr sheath. Thus, if a 6Fr sheath can be inserted without resistance, the 8Fr GC can be inserted. Additionally, from our experience, any GC from any manufacturer can be used.

The disadvantage of DCA was that an 8Fr GC, which is preferred for the TFA, was usually required, thus increasing the potential risk of bleeding complications and patient burden due to prolonged bed rest. Hence, the DCA procedure is often discouraged in an era when TRA is the mainstream. Although we did not assess the precise length of post-operative resting period, the insert pain of GC, patient’s pain complaints, and resting time were likely to be reduced. According to resting protocol of our institution, the patients of the TRA group are able to walk immediately after surgery, whereas patients of the TFA group are on bed rest for at least 5 h after surgery. Therefore, the clinical implication of this study is that DCA with TRA 8Fr sheathless GC can be more safely performed without increasing the VASC as well as potentially reducing resting time, thereby decreasing the patient burden.

While these findings support the feasibility of DCA with TRA using the 8Fr sheathless GC, several limitations must be acknowledged. First, it was a retrospective observational study, and the choice of vascular access (TRA vs. TFA) was not randomized but left to the discretion of the operators, introducing potential selection and reporting biases. Indeed, there was a tendency to select younger and heavier patients for the TRA group, likely due to favorable radial artery anatomy. As such, the 8Fr sheathless TRA system may not be appropriate for smaller or older patients, given the generally smaller diameter, increased tortuosity, and stiffness of the radial artery in this population. Furthermore, although not performed routinely in all patients, the ultrasound assessment of the radial artery to confirm vessel diameter and radial occlusion before catheterization may have been useful and helpful in identifying predictors of the feasibility of this method^16,17^. The complexity of coronary lesions was also lower in the TRA group, suggesting a possible operator tendency to avoid complex anatomies that might limit guiding catheter maneuverability. Additionally, while all operators were experienced in DCA, they had relatively limited experience with the 8Fr sheathless GC via the radial approach. Operator-specific variability could have influenced outcomes, although no significant differences were observed among the three participating centers. Lastly, this study did not include a priori sample size calculation, as it was retrospective and based on available clinical data. Therefore, although statistically significant differences were observed, the findings should be interpreted with caution.

Although only 26% of patients in this cohort underwent DCA with TRA using an 8Fr sheathless GC, the high technical success rate (80.4%) and absence of crossover suggest that this approach is both feasible and safe in appropriately selected cases. Based on our institutional experience and the favorable procedural outcomes observed in this series, we estimate that a larger proportion of patients undergoing DCA could be suitable candidates for this strategy as a primary access option, particularly when anatomical factors, such as radial artery caliber and lesion complexity, are favorable. While this estimation is not derived from a formal eligibility analysis, it reflects practical insights from clinical experience and highlights the potential for broader use. Future randomized controlled trials with larger cohorts, standardized access protocols, and comprehensive outcome evaluations are warranted to validate its generalizability and further refine patient selection criteria.

In conclusion, DCA with TRA using the 8Fr sheathless GC may represent a safer and equally feasible alternative to TFA for DCA. This technique demonstrated significantly lower access site complication rates while maintaining comparable procedural success, making it a promising option for reducing patient burden and improving procedural safety in appropriately selected cases.

## Data Availability

All data regarding this case has been reported in the manuscript. Please contact the corresponding author if you are interested in any further information.
